# Mode of action evaluation for reduced reproduction in *Daphnia pulex* exposed to the insensitive munition, 1-methyl-3-nitro-1-nitroguanidine (MeNQ)

**DOI:** 10.1007/s10646-021-02447-w

**Published:** 2021-06-26

**Authors:** Kurt A. Gust, Guilherme R. Lotufo, Natalie D. Barker, Qing Ji, Lauren K. May

**Affiliations:** 1 US Army, Engineer Research and Development Center, Environmental Laboratory, Vicksburg, MS 39180 USA; 2 Bennett Aerospace Inc, Cary, NC 27511 USA

**Keywords:** *Daphnia pulex*, Genomics, Reproductive Toxicology, 1-methyl-3-nitro-1-nitroguanidine (MeNQ), Insensitive Munitions

## Abstract

The US Department of Defense (DOD) is developing insensitive munitions (IMs) that are resistant to unintended detonation to protect warfighters. To enable material life-cycle analysis for the IM, 1-methyl-3-nitro-1-nitroguanidine (MeNQ), ecotoxicological impacts assessment was required. A previous investigation of MeNQ exposures in *Daphnia pulex* revealed concentration-responsive decreases in reproduction relative to controls (0 mg/L) across a 174, 346, 709, 1385, and 2286 mg/L exposure range. The present study used those exposures to conduct global transcriptomic expression analyses to establish hypothetical mode(s) of action underlying inhibited reproduction. The number of significantly affected transcripts and the magnitude of fold-change differences relative to controls tended to increase with increasing MeNQ concentration where hierarchical clustering analysis identified separation among the “low” (174 and 346 mg/L) and “high” (709, 1385, and 2286 mg/L) exposures. Vitellogenin is critical to *Daphnia* reproductive processes and MeNQ exposures significantly decreased transcriptional expression for vitellogenin-1 precursor at the lowest exposure level (174 mg/L) with benchmark dose (BMD) levels closely tracking concentrations that caused inhibited reproduction. Additionally, juvenile hormone-inducible protein, chorion peroxidase, and high choriolytic enzyme transcriptional expression were impacted by MeNQ exposure having potential implications for egg production / maturation and overall fecundity. In concert with these effects on specific genes involved in *Daphnia* reproductive physiology, MeNQ exposures caused significant enrichment of several canonical-pathways responsible for metabolism of cellular energy substrates where BMD levels for transcriptional expression were observed at ≤100 mg/L. These observations imply possible effects on whole-organism energy budgets that may also incur indirect costs on reproduction.

## Introduction

The US Department of Defense (DOD) has been required by law to replace conventional munitions with insensitive munitions (IMs) resistant to unintended detonation to protect the lives of warfighters. Among the chemicals suitable for use as constituents in IM formulations, 1-methyl-3-nitroguanidine (MeNQ) (Aubert and Roos 2014; Reinke 2016) has had relatively little characterization of potential health and ecotoxicological hazard. While the ecotoxicity of dinitroanisole (DNAN), nitroguanidine (NQ), and nitrotriazolone (NTO), the components of IMX-101, a principal TNT replacement, has been widely investigated (Lent et al. 2015, 2016, 2018; Lotufo et al. 2018; Gust et al. 2018, 2021; Johnson et al. 2017; Kennedy et al. 2015, 2017; Quinn et al. 2014; Stanley et al. 2015), only two studies describing MeNQ toxicity in mammalian exposures (Kinkead et al. 1993; Reinke 2016) and our laboratory’s three recent MeNQ ecotoxicological evaluations (Lotufo et al. 2020, 2021; Gust et al 2021) are the only studies presently available. In these ecotoxicology studies, the lethal effects of MeNQ in both acute and chronic aquatic exposures were only observed at high exposure concentrations (≥ 2186 mg/L) for a broad range of target species including both aquatic invertebrates (*Daphnia pulex, Chironomus dilutus*, *Lumbriculus variegatus*, *Hydra littoralis*, *Hyalella azteca*) and vertebrates (*Pimephales promelas*, *Rana pipiens*). However, investigation of sublethal effects on growth and reproduction in Lotufo et al. (2021) revealed increased sensitivity to MeNQ exposures in *D. pulex*, *C. dilutus*, and *H. azteca*, where *D. pulex* showed the greatest sublethal sensitivity to MeNQ exhibiting dose-responsive decreases in reproduction with significant effects initiated at the lowest exposure concentration tested (174 mg/L). Impacts on reproduction represent a critical adverse outcome in regulatory ecotoxicology posing a direct threat to population sustainability (Ankley et al. 2010; Eggen et al. 2004). Efforts to manage risk associated with reproductive impacts, such as those observed for MeNQ, require knowledge about how these reproductive impacts occur, where toxicological modes and mechanisms of action are needed to provide fundamental insight.

As a means to facilitate discovery of the toxicological mode(s) and potential mechanism(s) underlying the reproductive impacts of MeNQ exposures in *D. pulex*, we conducted global transcriptomic expression assays and analyses to identify critical genes and functional pathways affected by MeNQ exposure to connect molecular responses to reproductive outcomes. We have successfully applied this approach to generate hypothetical modes and mechanisms of action in previous munitions and IM investigations (Rawat et al. 2010; Gust et al. 2009, 2015, 2018, 2019a, 2021) in which many have been validated and transitioned into adverse outcome pathways (AOPs) for regulatory consideration (Wilbanks et al. 2014; Gong et al. 2015; Collier et al. 2016; Gust et al. 2019b). The present study utilized *D. pulex* samples taken directly from the chronic reproductive toxicity assay described in Lotufo et al. (2021) where transcriptomic expression was investigated using custom microarrays developed based on the sequenced *D. pulex* genome (Coulbourne et al. 2011). In addition to leveraging UniProt annotations for basic eukaryotic gene functions (Uniprot Consortium, 2018), effects on gene-transcripts representing the unique reproductive biology of *D. pulex* were also investigated using text mining for reproductive functions in *Daphnia*, as previously described in Gust et al (2019c). Overall, the present study provides evidence of MeNQ-induced changes in transcriptional expression for genes directly involved in *D. pulex* reproductive physiology as well as systems-level effects on energy metabolic processes that have logical connections to observed impacts on *D. pulex* reproduction.

## Materials and methods

The present study was initiated using *D. pulex* sampled at the termination of the three-brood chronic reproduction assay described in Lotufo et al. (2021) to conduct microarray-based global transcriptomic investigations. In the Lotufo et al. (2021) study, *D. pulex* were exposed to MeNQ at control (0 mg/L), 174, 346, 709, 1385, and 2286 mg/L (measured concentrations) as juveniles through three-broods of reproduction. This exposure caused reproductive inhibition, resulting in a 50% inhibitory concentration (IC50) of 424 mg/L with a 95% confidence interval (C.I) of (159–609 mg/L). Although, MeNQ has not yet been deployed in operational munitions platforms, concentrations of conventional munitions at blast targets on military training sites may have high soil concentrations where measurements in the 10 s, 100 s, and up to 1000 mg/kg have been observed (Thiboutot et al. 2004). Coupled with the high water solubility of MeNQ, the concentrations used represent plausible aquatic exposures, albeit at the highest limit of expected environmental exposures. The three-brood reproduction experiment was executed using modified Zumwalt boxes, as described in Laird et al. (2015), which included 10 replicate *D. pulex* individuals monitored for reproduction and reported in Lotufo et al. (2021). To increase the amount of *D. pulex* tissue available for transcriptomic analysis, the experiment size was doubled to 20 replicate individuals per treatment, which were all run simultaneously. The animals remaining at the termination of the assay were assigned to a set of 4 “transcriptomics replicate” groups of individuals per treatment to serve as treatment replicates in the transcriptomic-expression experimental design (6 treatments × 4 replicates, where each replicate included 3–5 individual daphnids). The *D. pulex* samples were flash frozen in liquid nitrogen and stored at −80 °C until RNA extractions were conducted. The sample set was utilized to conduct global transcriptomic expression analysis to identify hypothetical modes of action related to the decreased reproductive rates observed by Lotufo et al. (2021) in response to the MeNQ exposures.

### RNA extractions

Frozen samples were homogenized using disposable mortar and pellet pestles for each sample (Kimble Kontes, Vineland, NJ). Total RNA isolation and on-column DNase digestion was conducted using the Qiagen RNeasy Mini Kit (Qiagen, Germantown, MD) following manufacturer’s recommendations. RNA quantity was assessed using a NanoDrop One Spectrophotometer (NanoDrop technologies, Wilmington, DE, USA) where a minimum RNA quality measurement of ≥2 for the 28 S :18 S ribosomal RNA (rRNA) ratio was required for sample use. Final RNA quality was determined using an Agilent 2100 Bioanalyzer (Agilent Technologies, Waldbronn, Germany) where visual inspection of electrophoresis gels showed high-quality samples as demonstrated by sharp 28 S :18 S ribosomal rRNA bands and the expected minor smear of bands across the RNA size spectrum (Supplementary Table S1).

### Transcript expression experiments

The effects of MeNQ exposures on transcriptional expression in *D. pulex* were investigated using microarray assays. An Agilent Technologies (Agilent Technologies, Santa Clara, CA, USA) single color custom 8 x 60 K microarray format (Amadid #: 063815) was used for all investigations. A completely randomized design was utilized for microarray hybridizations where each individual *D. pulex* sample was selected at random (using a random number generator) for hybridization onto individual microarrays. The Agilent Low Input Quick Amp Labeling Kit (one color) and hybridization protocol (Agilent Technologies) were utilized for microarray hybridizations following manufacturer’s recommendations using 65 ng of total RNA as starting material from each biological sample. In summary, a total of 24 microarrays were hybridized which included four replicates for all MeNQ exposures including the control (0 mg/L), 174, 346, 709, 1385, and 2286 mg/L exposure treatments.

### Microarray analysis

An Agilent Technologies, High-Resolution Microarray Scanner (Model G2505C, Agilent Technologies, Santa Clara, CA, USA) was used to scan microarray images at 2 μm resolution. Data were extracted from microarray images using Agilent Feature Extraction software, version 10.7.3.1. (Agilent Technologies). Microarray data were normalized to the 75th percentile within each array followed by median scaling among all exposures using GeneSpring Software version GX 14.9 (Agilent Technologies). GeneSpring was also utilized for the expression analysis where a one-way ANOVA was conducted (*p* = 0.01) including Benjamini-Hochberg multiple-tests corrections (also at *p* = 0.01). Post-hoc tests were conducted where the SNK test was utilized to determine which transcripts had significant differential expression relative to the 0 mg/L control in addition to a minimum ±1.5 fold-change cutoff which was applied because interpretations for transcriptional fold changes less than ±1.5 can be difficult. Overall, the combination of the stringent statistical test (*p* = 0.01 with multiple-tests correction) and ±1.5 fold-change cutoff provides a conservative assessment of differentially-expressed transcripts erring on the side of eliminating false-positive gene identifications with the potential tradeoff of false negative exclusions. Finally, GeneSpring was used to visualize clustering of significant transcripts using 3-dimensional principal component analysis as well as hierarchical clustering analysis based on Euclidean distance and Wards linkage rules.

### Functional annotation

The database for annotation, visualization and integrated discovery (DAVID, version 6.8, Huang et al. 2009) was used to conduct statistical enrichment analysis where significantly enriched pathways were derived based on gene-transcripts that had significant differential expression within each exposure. Pathway enrichment tests were derived for the full suite of Kyoto Encyclopedia of Genes and Genomes (KEGG) pathways. UniProt IDs (UniProt Consortium, 2018) represent the primary functional annotations mapped to the *D. pulex* microarray probes and were thus used for the KEGG pathway enrichment analysis. The UniProt annotations for *D. pulex* represent orthologous gene matches to model species including primarily *Homo sapiens*, *Mus musculus*, *Drosophila melanogaster*, as well as other well described genomic models. Given that *Homo sapiens* annotations were the most abundant for all significant gene-sets analyzed, it was used as the “background” transcriptome for the enrichment analyses. The significantly enriched KEGG pathways were used to posit general functional responses likely to be conserved between *D. pulex* and human gene function, such as basic responses conserved across eukaryotes.

### Text mining of D. pulex protein annotations

Although the UniProt annotations provide value for gene functions conserved across eukaryotes, the *D. pulex* genome (Colbourne et al. 2011) includes a broad abundance of genes / gene functions unique to daphnids which is lost when only investigating orthology to distantly related species, such as mammals. To add value to the novel gene annotations within the *D. pulex* genome, text mining was conducted for all transcripts having significant differential expression in response to the MeNQ treatments using key words to identify putative gene functions connected to characteristic reproductive phenotypes in *Daphnia* species, an approach we have conducted successfully in a prior study of *Daphnia* (Gust et al. 2019c). Specifically, text mining was conducted for terms related to *Daphnia* reproduction, egg production, molting / cuticle processes (important for brood release), and etc, which are provided in Supplementary Table S2, to identify putative functional responses to the MeNQ exposure treatments that caused the reduced reproduction in *D. pulex* described in Lotufo et al. (2021).

### Dose-response testing and benchmark dose (BMD) calculations for transcriptional expression

The dose-response relationships among MeNQ exposures and transcriptional expression were investigated using the DRomics tool (http://lbbe-shiny.univ-lyon1.fr/DRomics/inst/DRomics-shiny/) to characterize and statistically test dose-response relationships and calculate benchmark dose (BMD) values (Larras et al. 2018). The analysis was run using the transcripts observed to have significant differential expression using the criteria described in the “Microarray analysis” section above. An array of dose-response models were deployed as part of the automated DRomic tool analysis where best fits were established as part of the analysis pipeline (Larras et al. 2018). A significant model fit was established using p = 0.05 for each transcriptional target. Benchmark dose (BMD) values were also calculated when significant model fits were identified. Two BMD values were calculated, the first being determined based on a point of departure for expression that varied 2 standard deviations from the control, “BMD (2 SD)”, and a point of departure based on a ≥ 1.5-fold change difference in expression relative to the control, “BMD (1.5-fold)”. To provide an overview of the BMD results, mean BMD values with associated standard errors were plotted for all transcriptional targets contributing to significantly enriched KEGG pathways in addition to the differentially-expressed reproduction-related transcripts identified in the text mining analysis.

## Results

### Transcriptomics expression overview

**Fig. 1:**
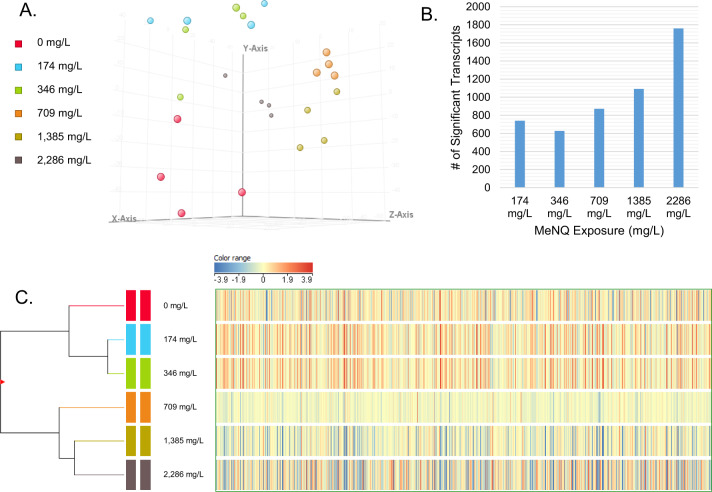
Summary diagnostics of transcript expression analysis for the *Daphnia pulex* exposures to MeNQ. **A** Principal component analysis of significant transcripts (Benjamini-Hochberg multiple-tests corrected, p = 0.01) where 53.1, 19.3, 8.7% of the variance is explained by principal components X, Y, and Z, respectively. **B** The total number of significant differentially expressed transcripts relative to the control for each MeNQ exposure level in SNK post-hoc pairwise tests. **C** Hierarchical clustering analysis using normalized intensity values of significant transcripts based on Euclidean distance and Wards linkage rules. The color range represents the normalized microarray signal data for all transcriptional targets

Microarray signal detection was achieved for 60,944 probes representing 30,472 unique transcriptional targets for *D. pulex*. Exposure to MeNQ caused significant changes in transcriptional expression of 2322 gene targets (Supplementary Table S3) where multiple-test corrections predicted a margin of 23 transcripts affected by chance. Principal component analysis of significantly affected transcripts displayed clear separation of experimental treatments from the control and clustering of treatment replicates by MeNQ exposure concentration (Fig. 1A). In pair-wise comparisons against controls, an increasing number of differentially expressed transcripts was observed with increasing exposure concentrations beyond the 346 mg/L exposure concentration (Fig. 1B). Finally, hierarchical clustering analysis of significant transcripts indicated a separation in expression patterns among the “low” and “high” MeNQ exposure concentrations where control, 174 mg/L, and 346 mg/L treatments clustered separately from the 709 mg/L, 1385 mg/L and 2286 mg/L exposures (Fig. 1C). All microarray data are available at the Gene Expression Omnibus (GEO) under series accession GSE164957.

### Functional pathway responses to MeNQ exposures

The MeNQ exposure caused significant enrichment of KEGG pathways (Table 1) which tended to sort by “low” and “high” exposure groups, in accordance with the separation observed in the hierarchical clustering analysis described in the previous section (Fig. 1C). The low MeNQ exposures caused significant enrichment of pathways involved in general energy metabolic processes including: starch and sucrose metabolism, fat digestion and absorption, alpha-linolenic acid metabolism, and ether lipid metabolism pathways (Table 1). Within these enriched pathways, primarily decreased transcriptional expression for genes within the starch and sucrose metabolism pathway (6 of 6 transcript targets) was observed, whereas expression was mixed (increased or decreased expression depending on gene target) for the fat digestion and absorption (3 targets decreased and 2 increased), the alpha-linolenic acid metabolism (2 increased and 2 decreased), and the ether lipid metabolism (3 targets decreased and 2 increased) pathways (Table 1, Supplementary Table S4). Significant enrichment of pathways involved in energy-metabolic pathways was also prominent in the high MeNQ exposure treatment set including: galactose metabolism, amino sugar and nucleotide sugar metabolism, pancreatic secretion, and protein digestion and absorption pathways (Table 1). Within this set of pathways, trends of primarily dose-responsive decreases in transcriptional expression were observed for the pancreatic secretion (17 of 21 transcript targets), and protein digestion and absorption pathways (14 of 17 transcript targets, Table 1, Supplementary Table S4). Finally, the generalized “metabolic pathways” (hsa01100) pathway set was significantly enriched across all MeNQ exposures examined (Table 1) where expression across transcriptional targets was mixed (30 increased and 44 decreased) with gene identities representative of a broad variety of metabolic functions (Supplementary Table S4).

**Table 1:** Results of KEGG pathway enrichment analysis (*p* = 0.05) for transcripts having significant differential expression in response to MeNQ exposures

MeNQ	KEGG ID	KEGG pathway	*p* value	Gene Count	Gene Targets Sig. Increased	Gene Targets Sig. Dereased
Low MeNQ Exposures						
174 mg/L	hsa00500	Starch and sucrose metabolism	0.0302	3	0	6
346 mg/L	hsa00500	Starch and sucrose metabolism	0.0219	3	0	4
709 mg/L	hsa00500	Starch and sucrose metabolism	0.0406	3	0	6
346 mg/L	hsa00565	Ether lipid metabolism	0.0390	3	2	3
174 mg/L	hsa00592	alpha-Linolenic acid metabolism	0.0179	3	2	2
346 mg/L	hsa00592	alpha-Linolenic acid metabolism	0.0129	3	2	2
174 mg/L	hsa04146	Peroxisome	0.0311	4	0	2
346 mg/L	hsa04146	Peroxisome	0.0198	4	0	2
709 mg/L	hsa04146	Peroxisome	0.0467	4	0	2
174 mg/L	hsa04975	Fat digestion and absorption	0.0411	3	2	3
346 mg/L	hsa04975	Fat digestion and absorption	0.0300	3	2	3
High MeNQ Exposures						
709 mg/L	hsa00052	Galactose metabolism	0.0341	3	1	2
1385 mg/L	hsa00052	Galactose metabolism	0.0409	3	1	2
1385 mg/L	hsa00520	Amino sugar and nucleotide sugar metabolism	0.0146	4	1	4
2286 mg/L	hsa00520	Amino sugar and nucleotide sugar metabolism	0.0054	5	3	3
1385 mg/L	hsa01130	Biosynthesis of antibiotics	0.0260	7	3	4
2286 mg/L	hsa01130	Biosynthesis of antibiotics	0.0001	13	4	9
709 mg/L	hsa03010	Ribosome	0.0102	6	3	0
1385 mg/L	hsa03010	Ribosome	0.0033	7	4	0
2286 mg/L	hsa03010	Ribosome	0.0039	8	4	0
1385 mg/L	hsa04142	Lysosome	0.0095	6	4	4
2286 mg/L	hsa04142	Lysosome	0.0020	8	9	4
709 mg/L	hsa04972	Pancreatic secretion	0.0125	5	1	10
1385 mg/L	hsa04972	Pancreatic secretion	0.0031	6	0	15
2286 mg/L	hsa04972	Pancreatic secretion	0.0121	6	1	12
1385 mg/L	hsa04974	Protein digestion and absorption	0.0146	5	2	12
2286 mg/L	hsa04974	Protein digestion and absorption	0.0412	5	2	9
Both High and Low MeNQ Exposures						
174 mg/L	hsa01100	Metabolic pathways	0.0002	23	9	25
346 mg/L	hsa01100	Metabolic pathways	0.0009	19	9	13
709 mg/L	hsa01100	Metabolic pathways	0.0046	22	11	17
1385 mg/L	hsa01100	Metabolic pathways	0.0299	21	13	13
2286 mg/L	hsa01100	Metabolic pathways	0.0000	36	29	21

Commonality of significantly enriched KEGG pathways tended to sort into “low” and “high” exposure groups, therefore the table is presented as enriched pathways found in low, high, and both low and high MeNQ exposures. Transcriptional gene target identities and fold change values within each pathway is provided in Supplementary Table S4

### Text mining for D. pulex reproductive functions

**Fig. 2:**
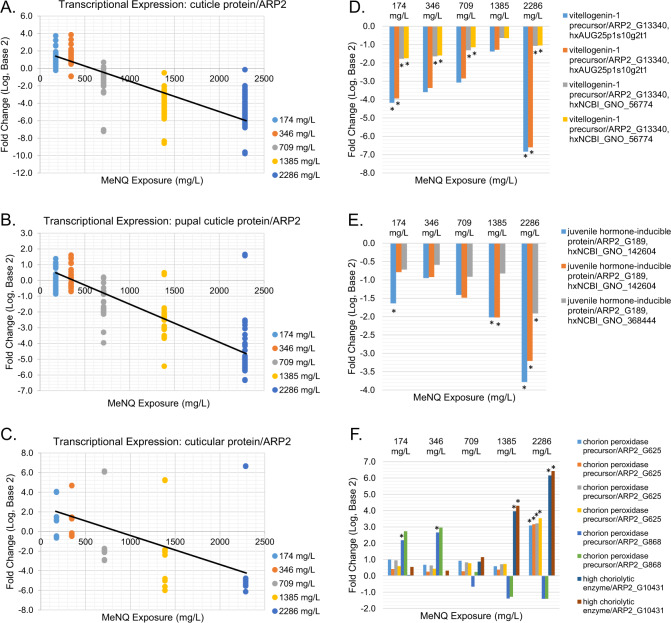
Transcriptional expression profiles for transcripts involved in reproduction and reproduction-related processes in *Daphnia pulex* which had significantly affected expression in response to MeNQ exposures. In **A**–**C**, the data points represent fold change values for all microarray targets representative of each specific gene. Significant (*p* = 0.05) linear regressions were identified for each of the 3 gene targets. In the bar charts (**D**–**F**), asterisks represent transcripts having significant differential expression relative to controls in one-way ANOVA with Benjamini-Hochberg multiple-test corrections (*p* = 0.01) and SNK pairwise *post-hoc* tests

The text mining of transcripts significantly affected in the MeNQ exposure that had contextual connections to *D. pulex* reproductive function provided multiple significant concentration-responsive outcomes in transcriptional expression (Supplementary Table S3, Fig. 2). This approach provided useful observations directly relevant to *Daphnia* reproductive biology. In Lotufo et al. (2021), which was leveraged as the *D. pulex* tissue source for the present study, a negative concentration-response relationship was observed where decreasing *D. pulex* reproduction corresponded with increasing MeNQ exposure concentration. In the present study, predominantly negative concentration-response relationships were observed between MeNQ exposure concentration and transcriptional expression for multiple representatives of the cuticle protein ARP2, pupal cuticle protein ARP2, and cuticular protein ARP2 (Fig. 2A–C, Supplementary Table S3). Multiple microarray targets coding vitellogenin-1 precursor had significantly decreased transcriptional expression in response to MeNQ, even at the lowest exposure concentration (Fig. 2D). Additionally, multiple array targets coding for juvenile hormone-inducible protein showed decreased transcriptional expression, the majority of which at the highest two exposure concentrations (Fig. 2E). Finally, the transcriptional expression of chorion peroxidase precursors showed unique concentration-response patterns based on the specific array target while transcriptional expression for the high choriolytic enzyme was significant increased at the highest two MeNQ exposure concentration (Fig. 2F).

### Dose-response relationships and benchmark dose (BMD) calculations

**Fig. 3:**
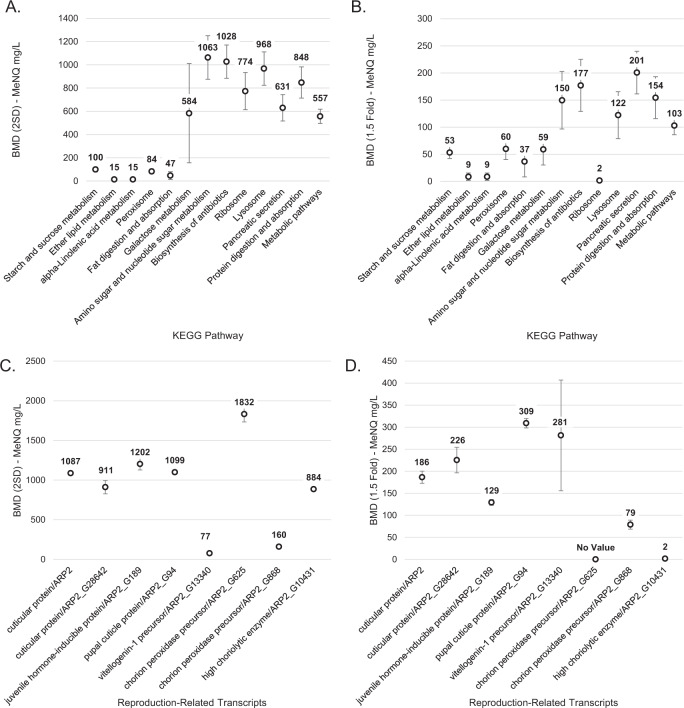
Benchmark dose (BMD) calculations summarized for all significantly enriched KEGG pathways (**A**, **B**) and the reproduction-related transcripts identified by text mining (**C**, **D**). Values represent mean BMD concentrations for transcriptional expression with error bars representing standard error. The “BMD (2 SD)” value represents the BMD based on 2 standard deviations of the control while “BMD (1.5 fold)” represents the BMD based on a 1.5-fold change difference in expression relative to the control

Significant dose-response model fits were identified for 2115 of the 2322 gene transcripts that had significant differential expression in response to the MeNQ exposure. A summary of the model-fit test results in addition to BMD calculations are provided in Supplementary Table S3 and results for all transcripts contributing to significantly enriched KEGG pathways are provided in Supplementary Table S4. The BMD values summarized for transcriptional expression within KEGG pathways ranged from 15–1063 mg/L for “BMD (2 SD)” and 9–201 mg/L for “BMD (1.5-fold)” (Fig. 3A and B). The higher relative values of BMD (2 SD) versus BMD (1.5 fold) are consistent with expectations for each BMD calculation type (Larras et al 2018), where the BMD (2 SD) represents the more conservative benchmark representative of a “critical” change at the theoretical upper or lower bound of the 95% coverage interval of the control (EFSA Committee et al. 2017). The results indicated that transcriptional expression of genes within the starch and sucrose metabolism, lipid metabolism, linolenic acid metabolism, peroxisome, and fat digestion and absorption KEGG pathways were sensitive to MeNQ exposure (Fig. 3A and B). The BMD calculations for transcripts involved in *Daphnia* reproductive-related processes (Fig. 3C and D) indicated BMDs ranging from 77–1832 mg/L and 2–309 mg/L for the BMD (2 SD) and BMD (1.5-fold) calculations, respectively. The BMD (2 SD) model, again, tended to provide more conservative values, however the value for vitellogenin-1 precursor was lower than was calculated using the BMD (1.5-fold) criteria.

## Discussion

The study by Lotufo et al. (2021), which served as the source of the *D. pulex* investigated in the present paper, demonstrated that exposure to MeNQ caused concentration-responsive decreases in *D. pulex* reproduction. Specifically, significant decreases in reproduction were observed relative to controls at all MeNQ concentrations investigated with ~50% reductions observed at 174 and 345 mg/L, ~65% reductions at 709 and 1385 mg/L, and ~100% reductions at 2286 mg/L. Summary values for reproductive inhibitory concentrations (IC) in *D. pulex* caused by the MeNQ exposures included an IC20 (20% reproductive inhibition) of 78 mg/L (64–140 mg/L, 95% C.I.) and an IC50 (50% reproductive inhibition) of 424 mg/L (159–609 mg/L, 95% C.I.). The transcriptomic expression analysis in the present paper provides multiple insights into the molecular responses to the MeNQ exposure that underlie this reproductive impact.

### MeNQ effects on gene-transcripts involved in reproductive processes

The *D. pulex* reproduction-focused text mining analysis provided multiple insights into potential modes and mechanisms of action underlying the reproductive impacts caused by the MeNQ exposure. First of which, the MeNQ exposure caused decreased transcriptional expression of vitellogenin-1 precursor in *D. pulex* (Figs. 2D, 3C and 3D). Vitellogenin is an egg yolk protein precursor which is an important contributor to oocyte production and egg quality in both invertebrate (Chang and Shih 1995) and vertebrate (Kime et al. 1999) species. In *Daphnia*, vitellogenin production originates in “fat cells” where transfer to oocytes occurs in the ovary (Zaffagnini and Zeni 1986). As has been observed in the freshwater amphipod, *Gammarus fossarum*, vitellogenin production plays a key role in female reproductive processes where vitellogenin quantity provides a key indicator of oocyte size and quality (Jubeaux et al. 2012). A variety of chemical contaminants including metals and organic compounds spanning diverse mechanisms of toxic action have been observed to negatively affect vitellogenin mRNA expression in *Daphnia magna* (Hannas et al. 2011), where contaminant exposures that reduce vitellogenin in *Daphnia* are an expected source of decreased overall reproductive success in chronic exposures (De Schamphelaere et al. 2004). In the present study, MeNQ exposure significantly reduced mRNA expression of vitellogenin-1 precursor across nearly every exposure concentration which corresponds with the reduced reproduction observed across all MeNQ exposure levels in the source animals (Lotufo et al. 2021). The lowest observed effect on transcriptional expression occurred at 174 mg/L (Fig. 2D) with BMDs calculated at 77 mg/L (BMD 2 SD) and 281 mg/L (BMD 1.5-fold), the lower of which closely tracking the 78 mg/L IC20 for reproduction observed in Lotufo et al. (2021). Based on these observations, it is plausible that the reduced transcriptional expression for vitellogenin precursors contributed to decreased *D. pulex* reproduction, if the reduced expression of mRNA translated into corresponding decreases in vitellogenin protein production.

Corresponding with the reduced transcriptional expression of vitellogenin-1 precursor, juvenile hormone-inducible protein also had decreased transcriptional expression in response to the MeNQ exposure and decreased dramatically with increasing MeNQ exposure concentration (Figs. 2E, 3C and 3D). Genomic investigations in *D. magna* have demonstrated a connection between certain vitellogenin-coding genes and proximally located juvenile hormone response elements, where experiments applying a juvenile hormone agonist caused reduced vitellogenin expression (Tokishita et al. 2006). Observations from the present study suggest that the reduced expression of vitellogenin-1 precursor is disconnected from the above-cited mechanism related to juvenile hormone-based regulation, given that transcriptional expression for both transcripts were decreased in response to MeNQ (Fig. 2E). Alternatively, application of the juvenile hormone analog (Altosid) in *D. magna* exposures inhibited embryonic development and production of viable offspring (Templeton and Laufer 1983). Additionally, juvenile hormone (also known as methyl farnesoate) has been observed to affect lipid storage / allocation processes in *Daphnia magna* causing the production of fewer, but larger offspring as well as an increased proportion of males, deviating from typical all-female broods expected in *D. magna* (Jordão et al. 2016). If these responses are conserved in *D. pulex*, decreased expression of juvenile hormone-inducible protein suggests decreased juvenile hormone signaling in response to MeNQ exposure with potential implications for embryonic development and lipid storage / allocation processes that may affect *Daphnia’s* reproductive success.

The MeNQ exposures also affected multiple gene targets coding various chorion peroxidase precursors where the ARP2_G625 and ARP2_G868 identities showed unique transcript expression trajectories (Fig. 2F). The lipid processing role of chorion peroxidase in *Daphnia magna* has been hypothesized to contribute to oogenesis (Fink and Windisch 2019), where chorion peroxidase is involved in a prostaglandin-processing pathway leading to vitellogenin production (Schlotz et al. 2016). The observations by Schlotz et al. (2016) lead that research group to hypothesize that chorion peroxidase activity in *D. magna* serves a similar role as observed in *Drosophila* where it provides an essential reproductive function (null mutants are sterile) in facilitating follicle maturation (Tootle and Spradling 2008). Transcriptional expression for high choriolytic enzyme, which acts as an egg hatching factor in fish (Yasumasu et al. 1989), showed a significant positive concentration-response relationship with MeNQ (Supplementary Table S3) with dramatic 16 to 86-fold increases in expression at the highest two exposure concentrations (Fig. 2F). Isolation of high choriolytic enzyme in the aquatic arthropods, *Artemia salina* and *Penaeus chinensis* showed choriolytic activity (Li et al. 2006, Fan et al. 2010) suggesting similar egg hatching function in these species, and thus, perhaps also in *Daphnia*. The transcriptional changes in chorion peroxidase and high choriolytic enzyme in MeNQ exposures are consistent with effects on egg development and physiology which may also have contributed to reduced reproduction in *D. pulex*, especially in light of potentially sensitive BMD relationships with MeNQ (Fig. 3).

Finally, the MeNQ exposure caused multiple significant dose-responsive (Supplementary Table S3), and in several cases, dramatic decreases in transcriptional expression (Fig. 2A–C) for a variety of cuticle and cuticular proteins in *D. pulex*. Cuticle proteins in *D. pulex* show similarity with insect orthologs that are involved in the molting cycle (Colbourne et al. 2007), where these molting functions in *D. magna* have been recently confirmed (Giraudo et al. 2017). The molt cycle for parental *Daphnia magna* has also been observed to have endocrine signaling connections to embryonic development in offspring with implications for reproductive success (Sumiya et al. 2016). Finally, transcriptional expression for cuticular proteins have been observed to vary with reproductive strategy in *Daphnia carinata* (Liu et al. 2014), therefore the known involvement of cuticle proteins in the molt cycle and probable connections to reproductive processes in *Daphnia* warrant a closer investigation of the largely dose-responsive decrease (Fig. 2 and Supplementary Table S3) in their transcriptional expression in response the MeNQ exposures.

### Effects on general and energy metabolic processes likely also contribute to reduced reproduction

Across all the MeNQ exposure concentrations, significant enrichment of the KEGG ontology, “metabolic pathways” (Table 1, Supplementary Table S4), was observed indicating that the MeNQ exposure elicited significant changes in transcriptional expression for 74 targets with gene identities incorporated across a variety of metabolic pathways, thus suggesting a potentially broadscale shift in homeostatic equilibrium in *D. pulex*. As a reminder, enrichment analysis of KEGG pathways was based on human annotations and pathways, therefore interpretation of these results should be evaluated based on the conservation of pathways among humans and *Daphnia* where more conserved pathways are expected to have more direct interpretive comparisons. Closer examination of specific statistically-enriched pathways revealed significant concentration-responsive decreases in transcriptional expression within pancreatic secretion, and protein digestion and absorption pathways, (Table 1, Supplementary Table S4). Additional pathways having significant enrichment in the MeNQ exposures included the starch and sucrose metabolism pathway which had significantly decreased transcriptional expression, as well as the fat digestion and absorption, ether lipid metabolism, alpha-linolenic acid metabolism, galactose metabolism, and amino sugar and nucleotide sugar metabolism pathways (Table 1, Supplementary Table S4), the majority of which showed relatively low BMD values, many ≤100 mg/L (Figs. 3A and 3B). A common thread among all of these enriched pathways is that each can play a role in regulating energy homeostasis, have it be through high-level signaling of energy metabolism processes, as associated with “pancreatic secretion process”, or through the metabolic processing of energy substrates by pathways such as the: starch and sucrose metabolism, protein digestion and absorption, and fat digestion and absorption pathways. In parallel to these observations, investigations of subacute MeNQ dosing in rats identified trends of reduced blood glucose and triglycerides relative to controls (Reinke 2016) indicating the potential for MeNQ exposures to negatively impact circulating energy substrates.

Changes in metabolic processes, especially those involved in the processing of cellular energy substrates, have important implications for an organism’s reproductive success. A large body of experimental, mathematical, and theoretical research has been devoted to understanding the utilization of cellular energy in *Daphnia* through the lens of dynamic energy budget (DEB) theory (Nisbet et al. 2000, 2010). Described briefly, DEB theory utilizes a central model which tracks energy assimilated from food versus energy expenditures for somatic maintenance (homeostasis), growth, maturity maintenance (developmental maturation), and reproduction. DEB models have also been deployed to understand the ecotoxicological outcomes of xenobiotic exposures, where observations by Ananthasubramaniam et al. (2014) have indicated that xenobiotic exposures tend to increase maintenance costs due to detoxification processes which ultimately reduce energy allocation to growth, maturation, and especially, reproduction. In the present study, the transcriptional evidence suggests that a variety of metabolic processes were likely affected by the MeNQ exposure, with many observations showing decreased expression for pathways involved in energy substrate processing, absorption, and metabolism (Table 1, Supplementary Table S4, Fig. 3). Further, the BMD values for altered expression within the several cellular-energy processing pathways, especially those occurring at exposure concentrations ≤100 mg/L of MeNQ (Fig. 3A and B) correspond with concentrations that elicited reproductive inhibition in *D. pulex* (Lotufo et al. 2021). If these transcriptional responses to MeNQ are indicative of changes at the metabolic level, it is plausible that cellular energy assimilation was negatively affected in *D. pulex*, thus reducing cellular energy availability for allocations to reproduction. As DEB models continue to proceed toward integration into quantitative adverse outcome pathways (qAOP) for growth and reproduction endpoints (Murphy et al. 2018), the utility of suborganismal data, including transcriptomics, to provide accurate integrative toxicological interpretations of xenobiotic impacts on these critical outcomes will continue to increase.

## Conclusions

We investigated the global transcriptomic responses underlying the observations reported by Lotufo et al. (2021) that aquatic exposures to the novel insensitive munition, MeNQ, caused decreased reproductive success in *D. pulex*. The transcriptomics results provided in the present study were used to establish hypothetical modes of action by which the MeNQ exposures inhibited *D. pulex* reproduction. Specifically, decreased transcriptional expression of a vitellogenin precursor and juvenile hormone-inducible protein in the MeNQ exposure, each gene a facilitator of reproductive physiology in *Daphnia*, have logical mechanistic connects to reduced offspring production (Fig. 1). Further, the BMD values for vitellogenin precursor expression (Fig. 3) closely tracked the MeNQ concentrations that inhibited reproduction. Additionally, the observations of affected transcriptional expression of chorion peroxidase and high choriolytic enzyme require closer investigation as possible contributors to impaired reproduction, given observed roles in arthropod egg production / maturation processes. The broadscale decreases in transcriptional expression for a diverse set of cuticle proteins observed in the MeNQ exposure additionally requires closer consideration regarding *Daphnia* embryo development and critical molting functions involved in brood release (Fig. 1), although BMD values for their expression may be at the high end or above the threshold of inhibited reproduction (Fig. 3). Finally, the significant enrichment of several pathways involved in general and especially energy metabolic processes in *D. pulex* require inspection through the lens of dynamic energy budget theory, where the increased metabolic costs of MeNQ detoxification processes and shifts in homeostatic equilibrium likely “spend” cellular energy that would otherwise be budgeted for reproduction. The BMD values for transcriptional expression of genes involved in cellular energy substrate metabolism pathways were affected at MeNQ concentration thresholds similar to concentrations that inhibited *D. pulex* reproduction (Fig. 3 and Lotufo et al 2021), thus these responses are likely indicative and/or involved in the broader mechanistic effects of the MeNQ-induced reduction in reproduction. Overall, the transcriptomic observations presented herein have provided a suite of hypothesized modes of action plausibly linked to impacted *D. pulex* reproduction. These modes of action should be tested in targeted assessments to determine how each contributes to the reduced reproduction observed in MeNQ exposures.

## Supplementary information

Supplementary Information

## Data Availability

The raw and processed microarray data is archived and available at the gene expression omnibus (GEO), https://www.ncbi.nlm.nih.gov/geo/ under series accession GSE164957. Additionally, results of the microarray analysis are available in the Supplementary Materials.

## References

[CR1] Ananthasubramaniam B, McCauley E, Gust KA, Kennedy AJ, Muller EB, Perkins EJ, Nisbet RM (2014). Relating suborganismal processes to ecotoxicological and population level endpoints using a bioenergetic model. Ecol Appl.

[CR2] Ankley GT, Bennett RS, Erickson RJ, Hoff DJ, Hornung MW, Johnson RD, Mount DR, Nichols JW, Russom CL, Schmieder PK, Serrrano JA, Tietge JE, Villeneuve DL (2010). Adverse outcome pathways: a conceptual framework to support ecotoxicology research and risk assessment. Environ Toxicol Chem.

[CR3] Aubert SA, Roos BD (2014) Melt cast insensitive eutectic explosive. U.S. Patent 8,663,406, issued March 4. https://patents.justia.com/patent/8663406.

[CR4] Chang C-F, Shih T-W (1995). Reproductive cycle of ovarian development and vitellogenin profiles in the freshwater prawns, *Macrobrachium rosenbergii*. Invert Reprod Dev.

[CR5] Colbourne JK, Eads BD, Shaw J, Bohuski E, Bauer DJ, Andrews J (2007). Sampling *Daphnia’s* expressed genes: preservation, expansion and invention of crustacean genes with reference to insect genomes. BMC Genomics.

[CR6] Colbourne JK, Pfrender ME, Gilbert D, Thomas WK, Tucker A, Oakley TH, Tokishita S, Aerts A, Arnold GJ, Basu MK, Bauer DJ, Cáceres CE, Carmel L, Casola C, Choi J-H, Detter JC, Dong Q, Dusheyko S, Eads BD, Fröhlich T, Geiler-Samerotte KA, Gerlach D, Hatcher P, Jogdeo S, Krijgsveld J, Kriventseva EV, Kültz D, Laforsch C, Lindquist E, Lopez J, Manak JR, Muller J, Pangilinan J, Patwardhan RP, Pitluck S, Pritham EJ, Rechtsteiner A, Rho M, Rogozin IB, Sakarya O, Salamov A, Schaack S, Shapiro H, Shiga Y, Skalitzky C, Smith Z, Souvorov A, Sung W, Tang Z, Tsuchiya D, Tu H, Vos H, Wang M, Wolf YI, Yamagata H, Yamada T, Ye Y, Shaw JR, Andrews J, Crease TJ, Tang H, Lucas SM, Robertson HM, Bork P, Koonin EV, Zdobnov EM, Grigoriev IV, Lynch M, Boore JL (2011). The ecoresponsive genome of *Daphnia pulex*. Science.

[CR7] Collier ZA, Gust KA, Gonzalez-Morales B, Gong P, Wilbanks MS, Linkov I, Perkins EJ (2016). A weight of evidence assessment approach for adverse outcome pathways. Regul Toxicol Pharmacol.

[CR8] De Schamphelaere KAC, Canli M, Van Lierde V, Forrez I, Vanhaecke F, Janssen CR (2004). Reproductive toxicity of dietary zinc to *Daphnia magna*. Aquat Toxicol.

[CR9] Hardy A, Benford D, EFSA Committee (2017). Update: use of the benchmark dose approach in risk assessment. EFSA Journal.

[CR10] Eggen RI, Behra R, Burkhardt-Holm P, Escher BI, Schweigert N (2004). Challenges in ecotoxicology. Environ Sci Technol.

[CR11] Fan T, Wang J, Yuan W, Zhong Q, Shi Y, Cong R (2010). Purification and characterization of hatching enzyme from brine shrimp *Artemia salina*. Acta Biochimica et Biophysica Sinica.

[CR12] Fink P, Windisch HS (2019). The essential omega-3 fatty acid EPA affects expression of genes involved in the metabolism of omega-6-derived eicosanoids in *Daphnia magna*. Hydrobiologia.

[CR13] Giraudo M, Douville M, Cottin G, Houde M (2017). Transcriptomic, cellular and life-history responses of *Daphnia magna* chronically exposed to benzotriazoles: Endocrine-disrupting potential and molting effects. PLoS One.

[CR14] Gong P, Hong H, Perkins EJ (2015). Ionotropic GABA receptor antagonism-induced adverse outcome pathways for potential neurotoxicity biomarkers. Biomark Med.

[CR15] Gust KA, Indest KJ, Lotufo G, Everman SJ, Jung CM, Ballentine ML, Hoke AV, Sowe B, Gautam A, Hammamieh R, Ji Q, Barker ND (2021). Genomic investigations of acute munitions exposures on the health and skin microbiome composition of leopard frog (*Rana pipiens*) tadpoles. Environ Res.

[CR16] Gust KA, Lotufo GR, Thiyagarajah A, Barker ND, Ji Q, Marshall K, Wilbanks MS, Chappell P (2019). Molecular evaluation of impacted reproductive physiology in fathead minnow testes provides mechanistic insights into insensitive munitions toxicology. Aquat Toxicol.

[CR17] Gust KA, Wilbanks MS, Collier ZA, Burgoon LD, Perkins EJ (2019b) Adverse Outcome Pathway on antagonist binding to PPARα leading to body-weight loss. OECD Series on Adverse Outcome Pathways No. 10. 10.1787/29d4e08d-en

[CR18] Gust KA, Kennedy AJ, Laird JG, Wilbanks MS, Barker ND, Guan X, Melby NL, Burgoon LD, Kjelland ME, Swannack TM (2019). Different as night and day: Behavioural and life history responses to varied photoperiods in *Daphnia magna*. Mol Ecol.

[CR19] Gust KA, Lotufo GR, Stanley JK, Wilbanks MS, Chappell P, Barker ND (2018). Transcriptomics provides mechanistic indicators of mixture toxicology for IMX-101 and IMX-104 formulations in fathead minnows (*Pimephales promelas*). Aquat Toxicol.

[CR20] Gust KA, Nanduri B, Rawat A, Wilbanks MS, Ang CY, Johnson DR, Pendarvis K, Chen X, Quinn MJ, Johnson MS, Burgess SC, Perkins EJ (2015). Systems toxicology identifies mechanistic impacts of 2-amino-4,6-dinitrotoluene (2A-DNT) exposure in Northern Bobwhite. BMC Genomics.

[CR21] Gust KA, Nanduri B, Rawat A, Wilbanks MS, Ang CY, Johnson DR, Pendarvis K, Chen X, Quinn MJ, Johnson MS, Burgess SC, Perkins EJ (2015). Systems toxicology identifies mechanistic impacts of 2-amino-4,6-dinitrotoluene (2A-DNT) exposure in Northern Bobwhite. BMC Genomics.

[CR22] Hannas BR, Wang YH, Thomson S, Kwon G, Li H, LeBlanc GA (2011). Regulation and dysregulation of vitellogenin mRNA accumulation in daphnids (*Daphnia magna*). Aquat Toxicol.

[CR23] Huang DAW, Sherman BT, Lempicki RA (2009). Systematic and integrative analysis of large gene lists using DAVID bioinformatics resources. Nat Protoc.

[CR24] Johnson MS, Eck WS, Lent EM (2017). Toxicity of insensitive munition (IMX) formulations and components. Propellants, Explosives, Pyrotechnics.

[CR25] Jordão R, Garreta E, Campos B, Lemos MFL, Soares AMVM, Tauler R, Barata C (2016). Compounds altering fat storage in *Daphnia magna*. Sci Total Environ.

[CR26] Jubeaux G, Simon R, Salvador A, Quéau H, Chaumot A, Geffard O (2012). Vitellogenin-like proteins in the freshwater amphipod *Gammarus fossarum* (Koch, 1835): Functional characterization throughout reproductive process, potential for use as an indicator of oocyte quality and endocrine disruption biomarker in males. Aquat Toxicol.

[CR27] Kennedy AJ, Poda AR, Melby NL, Moores LC, Jordan SM, Gust KA, Bednar AJ (2017). Aquatic toxicity of photo-degraded insensitive munition 101 (IMX-101) constituents.. Environ Toxicol Chem.

[CR28] Kennedy AJ, Laird JG, Lounds C, Gong P, Barker ND, Brasfield SM, Russell AL, Johnson MS (2015). Inter- and intraspecies chemical sensitivity: a case study using 2,4-dinitroanisole. Environ Toxicol Chem.

[CR29] Kime DE, Nash JP, Scott AP (1999). Vitellogenesis as a biomarker of reproductive disruption by xenobiotics. Aquaculture.

[CR30] Kinkead ER, Wolfe RE, Salins SA, Godin CS, Lu PP, Ketcha MM, Thilagar A, Brashear WT (1993). N-methyl-N’-mitroguanidine: Irritation, sensitization, and acute oral toxicity, genotoxicity, and methods for analysis in biological samples. Toxicol Ind Health.

[CR31] Laird JG, Kennedy AJ, Melby NL, Lounds C, Gong P (2015). Development of a chronic toxicity testing method for Daphnia pulex.

[CR32] Larras F, Billoir E, Baillard V, Siberchicot A, Scholz S, Wubet T, Tarkka M, Schmitt-Jansen M, Delignette-Muller ML (2018). DRomics: a turnkey tool to support the use of the dose–response framework for omics data in ecological risk assessment. Environ Sci Technol.

[CR33] Lent EM, Mullins AB, May AD, Honnold CL, Despain KE (2018). Characterization of the testicular toxicity of 3-Nitro-1,2,4-Triazol-5-One and 2,4-dinitroanisole in rats (*Rattus norvegicus*). Int J Toxicol.

[CR34] Lent EM, Crouse LC, Wallace SM (2016). Oral toxicity of 2,4-dinitroanisole in rats. Int J Toxicol.

[CR35] Lent EM, Crouse LC, Wallace SM, Carroll EE (2015). Peri-pubertal administration of 3-nitro-1,2,4-triazol-5-one (NTO) affects reproductive organ development in male but not female Sprague Dawley rats. Reprod Toxicol.

[CR36] Li B-J, Fan T-J, Yang L-L, Cong R-S, Li L, Sun W-J, Lu C-X, Shi Z-P (2006). Purification and characterization of hatching enzyme from shrimp *Penaeus chinensis*. Arch Biochem Biophys.

[CR37] Liu A, Zhang M, Kong L, Wu D, Weng X, Wang D, Zhao Y (2014). Cloning and expression profiling of a cuticular protein gene in *Daphnia carinata*. Dev Genes Evol.

[CR38] Lotufo GR, Gust KA, Ballentine ML, Moores LC, Kennedy AJ, Barker ND, Ji Q, Chappell P (2020). Comparative toxicological evaluation of UV-degraded versus parent-insensitive munition compound 1-methyl-3-nitroguanidine in fathead minnow. Environ Toxicol Chem.

[CR39] Lotufo GR, Ballentine ML, May LR, Moores LC, Gust KA, Chappell P (2021) Multi-species Aquatic Toxicity Assessment of 1-Methyl-3-Nitroguanidine (MeNQ). Arch Environ Contam Toxicol. 10.1007/s00244-020-00796-x 10.1007/s00244-020-00796-x 33386940

[CR40] Lotufo GR, Stanley JK, Chappell P, Melby NL, Wilbanks MS, Gust KA (2018). Subchronic, chronic, lethal and sublethal toxicity of insensitive munitions mixture formulations relative to individual constituents in *Hyalella azteca*. Chemosphere.

[CR41] Murphy CA, Nisbet RM, Antczak P, Garcia-Reyero N, Gergs A, Lika K, Mathews T, Muller EB, Nacci D, Peace A, Remien CH, Schultz IR, Stevenson LM, Watanabe KH (2018). Incorporating suborganismal processes into dynamic energy budget models for ecological risk assessment. Integr Environ Assess Manag.

[CR42] Nisbet RM, McCauley E, Johnson LR (2010). Dynamic energy budget theory and population ecology: lessons from *Daphnia*. Philosoph Trans R Soc Lond Ser B, Biolog Sci.

[CR43] Nisbet RM, Muller EB, Lika K, Kooijman SALM (2000). From molecules to ecosystems through dynamic energy budget models. J Anim Ecol.

[CR44] Quinn MJ, Bannon DI, Jackovitz AM, Hanna TL, Shiflett AA, Johnson MS (2014). Assessment of 3-nitro-1,2,4-triazol-5-one as a potential endocrine disrupting chemical in rats using the Hershberger and uterotrophic bioassays. Int J Toxicol.

[CR45] Rawat A, Gust KA, Deng Y, Garcia-Reyero N, Quinn MJ, Johnson MS, Indest KJ, Elasri MO, Perkins EJ (2010). From raw materials to validated system: the construction of a genomic library and microarray to interpret systemic perturbations in Northern bobwhite. Physiological genomics.

[CR46] Reinke EN (2016) Effects of Acute and subacute oral methylnitroguanidine (MeNQ) exposure to rats (*Rattus norvegicus*). Toxicology Study No. S.0024883. 2016. Army Public Health Center (Provisional) Aberdeen Proving Ground United States.

[CR47] Schlotz N, Roulin A, Ebert D, Martin-Creuzburg D (2016). Combined effects of dietary polyunsaturated fatty acids and parasite exposure on eicosanoid-related gene expression in an invertebrate model. Comp Biochem Physiol Part A: Mol Integr Physiol.

[CR48] Stanley JK, Lotufo GR, Biedenbach JM, Chappell P, Gust KA (2015). Toxicity of the conventional energetics TNT and RDX relative to new insensitive munitions constituents DNAN and NTO in *Rana pipiens* tadpoles. Environ Toxicol Chem.

[CR49] Sumiya E, Ogino Y, Toyota K, Miyakawa H, Miyagawa S, Iguchi T (2016). Neverland regulates embryonic moltings through the regulation of ecdysteroid synthesis in the water flea *Daphnia magna*, and may thus act as a target for chemical disruption of molting. J Appl Toxicol.

[CR50] Templeton NS, Laufer H (1983). The effects of a juvenile hormone analog (Altosid ZR-515) on the reproduction and development of *Daphnia magna* (Crustacea: Cladocera). Int J Invertebrate Reprod.

[CR51] Thiboutot S, Ampleman,G, Marois A, Gagnon A, Bouchard M, Hewitt A, Jenkins T, Walsh M, Bjella K, Ramsey C, Ranney TA (2004) Environmental Conditions of Surface Soils, CFB Gagetown Training Area: Delineation of the Presence of Munitions Related Residues (Phase 3, Final Report). Defense R&D Canada –Valcartier, pp. 1–109. Technical Report, TR 2004-205.

[CR52] Tokishita S-i, Kato Y, Kobayashi T, Nakamura S, Ohta T, Yamagata H (2006). Organization and repression by juvenile hormone of a vitellogenin gene cluster in the crustacean, *Daphnia magna*. Biochem Biophys Res Commun.

[CR53] Tootle TL, Spradling AC (2008). *Drosophila* Pxt: a cyclooxygenase-like facilitator of follicle maturation. Development.

[CR54] Uniprot Consortium (2018). UniProt: a worldwide hub of protein knowledge. Nucleic Acids Res.

[CR55] Wilbanks MS, Gust KA, Atwa S, Sunesara I, Johnson D, Ang CY, Meyer SA, Perkins EJ (2014). Validation of a genomics-based hypothetical adverse outcome pathway: 2,4-dinitrotoluene perturbs PPAR signaling thus impairing energy metabolism and exercise endurance. Toxicol Sci.

[CR56] Yasumasu S, Iuchi I, Yamagami K (1989). Purification and partial characterization of high choriolytic enzyme (HCE), a component of the hatching enzyme of the teleost, *Oryzias latipes*1. J Biochem.

[CR57] Zaffagnini F, Zeni C (1986). Considerations on some cytological and ultrastructural observations on fat cells of *Daphnia* (Crustacea, Cladocera). Bollettino di zoologia.

